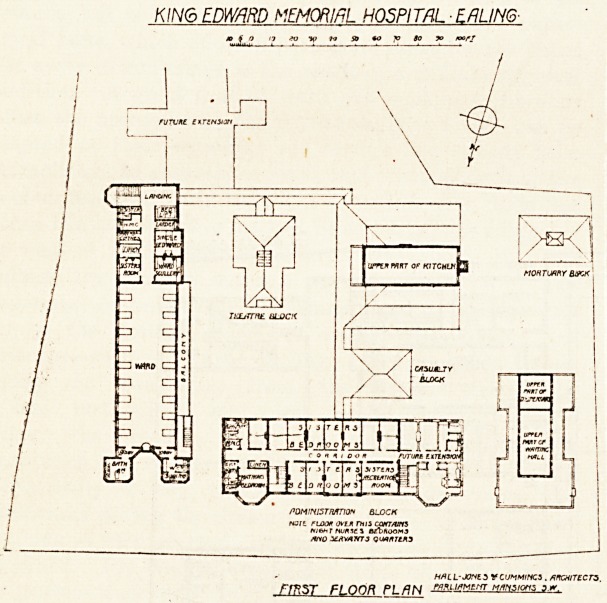# Hospital Architecture and Construction

**Published:** 1911-11-11

**Authors:** 


### HOSPITAL ARCHITECTURE AND CONSTRUCTION.

[Communications on this subject should be marked "Architecture" in the left-hand top corner of the envelope.]

### King Edward Memorial Hospital, Ealing.

THE site upon which this hospital is built is a very confined one, and not at all well adapted for the purpose. The consequence is that the arrangement of the building is cramped in a way that need not have been the case had the promoters of the hospital acquired a more suitable site.

The hospital, as at present completed, comprises six separate blocks, one of which, the dispensary, is entirely isolated, and another, the casualty block, though attached by a covered way, has no communication with it, and is therefore practically isolated. The front administration block, the kitchen block, and the operation theatre block are connected by means of a covered way open at the sides; and the ward block and operating block are connected by a closed-in corridor.

The front administration block is incomplete, the portion to the west corresponding to that containing the resident medical officer's quarters being deferred for future erection. When this is built the matron will have a suite of rooms corresponding to that provided for the resident medical officer. The accommodation provided in this block comprises the usual official quarters, with bedrooms for nurses and servants in the two upper floors.

The dispensary building is practically an outpatient department reduced to its smallest terms. Two consulting-rooms, each with an examining-room, a waiting hall, dispensary, drug store and cash office complete the whole of the accommodation provided.

The casualty block, which we should have thought would have more conveniently formed part of the dispensary block, is placed between the front administration block and the kitchen block, and no provision is made for driving an ambulance up to the door.

The block contains two rooms and a bath-room and w.c., but no provision is made for a ward in which to keep a dying patient, or one who cannot for any reason be discharged or sent up to a ward. The kitchen block is well arranged as a whole, though the provision of larders would be adequate for a hospital many times the size of this. The position is a central one between the wards and the administration, an arrangement which makes for economy of service; but the open-sided covered way is an arrangement which is open to objection, and the necessity for which is not apparent.

KING EDWARD MEMORIAL HOSPITAL · EALING

FIRST FLOOR PLAN — HALL-JONES & CUMMINGS, ARCHITECTS, PARLIAMENT MANSIONS S.W.

The operation-room block is arranged very much in the way that has been adopted at other hospitals. The door to the *x*-ray room is placed within the outer doorway to the corridor, which is a grave mistake if it is intended to use the *x*-ray room for outside patients. In that case the entrance should be carefully cut off from the theatre corridor.

The ward block is a long straight block of two storeys in height, having on each floor a main ward for eighteen beds and a side ward for one bed, with the usual offices. The main wards have each only one fireplace; it must, therefore, be assumed that dependence is placed on some form of warming by radiators, presumably of hot water. This plan is, in our judgment, a bad one; the direct radiant heat from an open fireplace is healthier and pleasanter than radiation from hot iron; and the value of open fireplaces as exhaust ventilators is very great indeed. Both the staircase and the lift are inside the block, the lift having a separate shaft. It would have been better to have planned the staircase with a well-hole sufficiently large to take the lift, and to have separated both from the ward block by a cross ventilated bridge. As it is, when the future pavilion is built the staircase and lift shaft will form conduits for the passage of air from all the wards.

It seems to us that a great mistake has been made by the promoters of this hospital in building on so confined a site and on a scale which prevents the development of the institution in the future into a large general hospital suitable for the needs of the large and growing town it is intended to serve.

The architects for the hospital are Messrs. Hall-Jones and Erskine S. Cummings, of Ealing and London.

## Figures and Tables

**Figure f1:**
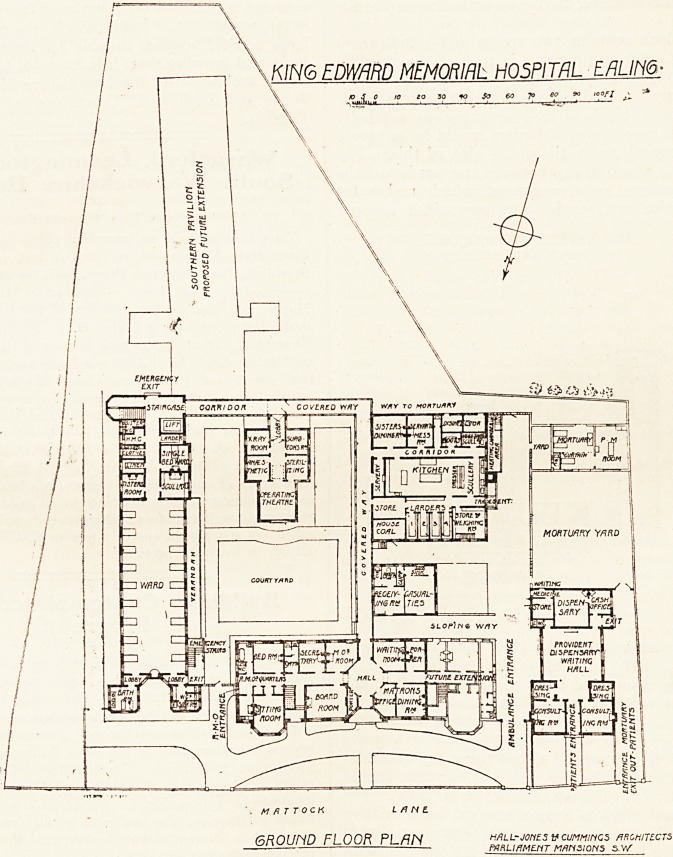


**Figure f2:**